# Accuracy and Reliability of AG501 Articulograph for Mandibular Movement Analysis: A Quantitative Descriptive Study

**DOI:** 10.3390/s20216324

**Published:** 2020-11-06

**Authors:** María Florencia Lezcano, Fernando Dias, Alain Arias, Ramón Fuentes

**Affiliations:** 1 Research Centre for Dental Sciences CICO, Department of Integral Adults Dentistry, Dental School, Universidad de La Frontera, Temuco 4811230, Chile; florencia.lezcano@ufrontera.cl (M.F.L.); fernando.dias@ufrontera.cl (F.D.); alain.arias@ufrontera.cl (A.A.); 2 Laboratorio de Cibernética, Departamento de Bioingeniería, Facultad de Ingeniería, Universidad Nacional de Entre Ríos, Oro Verde 3100, Argentina

**Keywords:** dentistry, biomechanics, electromagnetic articulography, mandibular movements

## Abstract

Electromagnetic articulography (EMA) have been mostly employed to study articulatory movements of speech. This technology appears to be very promising for studying mandibular movements within the field of dentistry. However, there are no studies reporting the validity of EMA for such purpose. The aim of this study is to assess accuracy and reliability of Carstens three-dimensional EMA AG501 in order to validate its use for mandibular movement analysis in dentistry. A set of tests was conducted attaching 16 sensors to a rotating rigid structure placed inside the measurement area. Another set of tests were conducted using a mouth anatomical model with human-like articulatory behaviour. A function of the EMA system called “head correction” was applied to normalize the data of every recording. The system reliability was higher at the centre of the measurement area and decreased toward the edges. Dispersion was greater for raw data than for normalized data. Bland-Altman analysis of agreement between the AG501 and a millimetre ruler used to measure the distance between the sensors revealed limits of agreement between 0.5 mm and −0.9 mm. The results suggest that EMA AG501 is valid for three-dimensional analysis of mandibular biomechanics allowing natural movements.

## 1. Introduction

Electromagnetic articulography (EMA) was described in the early 1970s as a research tool in speech physiology [1]. Its operating principle is based on the inductive measurement of distances and was originally applied to track the movement of articulators during speech production (tongue, lips, lower jaw) [1]. All EMA systems are similarly constructed, with multiple transmitter coils, which generate an alternating magnetic field of controlled frequency in the measurement area [1,2,3]. The magnetic field induces an alternating electric current in small receiver coils called sensors, and the induced current is processed as a signal in order to calculate the spatial position of the sensors [1].

The accuracy of EMA is determined by the degree of complexity and refinement of the calculation method derived from the application of the electromagnetic induction principles and also by the design and construction of the systems [1,2]. Initially, the recording capacity was limited by several factors, such as number and quality of transmitter/receiver coils, sensor positioning (initially restricted to one plane), calibration method, and distance-calculation method [1]. Over time, these limitations were overcome by the development of a new generation of EMA systems, among which was the AG series developed by Carstens and the Aurora/Wave developed by Northern Digital Instruments.

Several studies have compared the accuracy and reliability of articulographs to ensure valid speech articulatory movement recordings. Some reports have tested the functions of these systems in controlled laboratory environments to find component failures and sources of signal interferences and to check calculation methods [4,5,6,7,8,9,10]. Recently, Savariaux et al. compared the Wave, AG200, AG500, and AG501 articulographs and concluded that all systems can be considered suitable for speech articulatory measurements [8].

EMA systems have been mostly employed to study speech production and the act of swallowing [11,12,13,14,15,16,17]. In dentistry, mandibular movement analysis using EMA has been poorly developed, and there are no studies reporting the validity of EMA for such a purpose. Fuentes et al. employed the AG501 articulograph to assess biomechanical characteristics of masticatory cycles in healthy participants with normal occlusion [18,19,20]. Hoke et al. employed the Wave articulograph to determine dental prosthesis micro-movements during mastication [21]. These studies explored the potential of EMA systems applied to research in dentistry for the first time, but it is necessary to support their findings with the proper validation of the equipment employed. The aim of this study is to assess the accuracy and reliability of Carstens three-dimensional (3D) EMA AG501 in order to validate its use in mandibular movement analysis.

## 2. Materials and Methods

This quantitative descriptive study consisted in the analysis of thirty samples from each test of a series of tests (Table 1) carried out as follows.

Two experimental tests were conducted to assess the accuracy and reliability of Carstens 3D electromagnetic articulograph AG501. Both tests involved the use of a rigid structure attached to a rotating piece of the articulograph.

Three additional tests were conducted to evaluate the performance of the AG501 in mandibular movement analysis, for which a mouth anatomical model with human-like articulatory behaviour was employed. For every test, EMA sensors HQ220-L120-B (Carstens Medizinelektronik, Bovenden, Germany) were employed.

EMA raw data were normalized employing a specific function of the EMA system called “Head Correction,” which transforms raw coordinates into normalized coordinates relative to the position of the three reference sensors. All Cartesian coordinates registered with the EMA, raw and normalized, were processed using custom-developed Matlab scripts in order to obtain intelligible data representation and statistical results.

All the tests were conducted in the Oral Physiology Laboratory of the Research Centre in Dental Sciences, Universidad de La Frontera (Temuco, Chile).

### 2.1. Rotating Structure Tests

**Figure 1:**
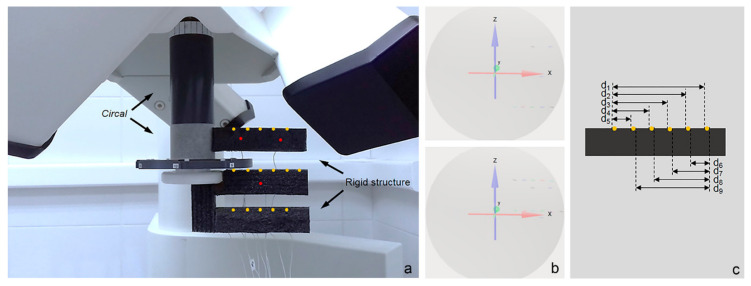
(**a**) Rigid structure attached to the circal and placement of sensors (active sensors, yellow; reference sensors, red); (**b**) position of the sensors (small arrows) inside the measurement area of the system (shaded area); (**c**) distances between sensors measured with a millimetre ruler (only one of the three levels of the structure showed).

Carstens articulograph AG501 has a rotating piece employed to calibrate the sensors called circal, which is located above the measurement area (Figure 1a). A rigid structure attached to the circal and placed inside the measurement area of the system (Figure 1a) was employed to conduct one static and one dynamic test. For both tests, 16 active sensors were glued to the structure on three vertical levels, as shown in (Figure 1a). Those three levels corresponded to z = −77, z = 0, and z = 77 of the system (Figure 1c). The distances between sensors (Figure 1b) were measured employing a millimetre ruler because it is the most commonly used instrument in dental clinical practice and the measurement will be used later to evaluate agreement between methods. Three reference sensors (Figure 1a) were placed to be able to normalize the data.

#### 2.1.1. Rotating Structure Static Test

Twenty-four recordings of the static position of the sensors glued to the structure were registered. Between each recording, the structure was rotated 15° counterclockwise in order to complete a 360° rotation. Thirty samples from every recording were included in the analysis.

#### 2.1.2. Rotating Structure Dynamic Test

Thirty recordings of a 360° rotation of the structure were registered. The rotation was carried out manually at an angular speed of approximately 80° per second.

### 2.2. Mouth Anatomical Model Tests

**Figure 2:**
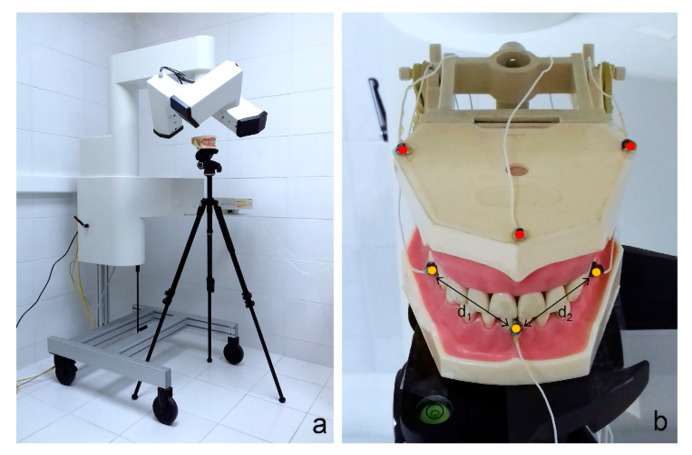
(**a**) Articulograph, tripod, and mouth anatomical model configuration; (**b**) placement of sensors on the mouth anatomical model (active sensors, yellow; reference sensors, red).

A mouth anatomical model attached to a tripod with 360° panning and dual-axis tilt (Figure 2a) was employed to conduct one static and two dynamic tests. Six sensors were glued on the model, as shown in (Figure 2b).

Every position of the anatomical model during the tests was restricted within the following extreme angles according to the possible head movements in healthy subjects summarized by Youdas [22]: Extreme flexion on the sagittal plane (70° Flex), extreme extension (90° Ext), extreme left flexion (78° LF), extreme right flexion (78° RF), extreme left rotation (78° LR), and extreme right rotation (78° RR).

#### 2.2.1. Mouth Anatomical Model Static Test

The static position of all sensors was registered for seven different orientations of the anatomical model: Upright position of the head with the model looking to the front (0°), 70° Flex, 90° Ext, 78° LF, 78° RF, 78° LR, and 78° RR. Thirty samples from every recording were included in the analysis.

#### 2.2.2. Mouth Anatomical Model Dynamic Test: Head Rotation, Flexion, and Extension

Head rotation, flexion, and extension movements were registered. All movements were performed manually rotating the tripod joint and reaching the angles mentioned before, according to the possible head movements in healthy subjects. Thirty repetitions of each movement were recorded.

#### 2.2.3. Mouth Anatomical Model Mouth Opening Test

Thirty repetitions of mouth opening starting from the maximal intercuspation position were registered for every orientation adopted in the mouth anatomical model static test described above.

### 2.3. Data Processing and Statistical Analysis

Descriptive statistics were implemented to analyze reliability. A Bland-Altman analysis of agreement between EMA and the millimeter ruler was implemented to evaluate accuracy.

#### 2.3.1. Standard Deviation (SD) Analysis (Rigid Structure Tests Data)

For all 24 angular positions adopted by the rigid structure in the static test, the Euclidean distance d_0_ between the origin of coordinates of the system and each sensor was calculated applying Equation (1).

(1) $$d_{o}=\sqrt{x^{2}+y^{2}+z^{2}}$$

The point defined by the coordinates (x, y, z) corresponds to the instantaneous position of one specific sensor. An SD of *d_o_* (*SD_do_*) was calculated to assess the accuracy of the EMA for different sectors of the measurement area applying Equations (2) and (3). Thirty (*n* = 30) consecutive samples from every recording were included.

(2) $$\bar{d_{o}}=\frac{\sum_{i=1}^{n}d_{oi}}{n}$$

(3) $$SD_{do}=\sqrt{\frac{\sum_{i=1}^{n}{\left(d_{o}-\bar{d_{o}}\right)}^{2}}{n-1}}$$

The same procedure was carried out for the dynamic test data, including also a total of 30 samples for every angular position. The number of samples included in this analysis was limited by the 30 repetitions of the dynamic test since the structure adopted each angular position only once for repetition. 

An analysis of EMA distance-measurement SD was implemented to evaluate EMA reliability when used to determine distances between sensors. Thirty samples (*n* = 30) of every angular position registered were used to calculate distances (*d*) and distances mean ($\bar{d}$) to arrive at the SD of distances (*SD_d_*) applying Equations (4) and (6).

(4) $$d=\sqrt{{\left(x_{1}-x_{2}\right)}^{2}+{\left(y_{1}-y_{2}\right)}^{2}+{\left(z_{1}-z_{2}\right)}^{2}}$$

(5) $$\bar{d}=\frac{\sum_{i=1}^{n}d_{i}}{n}$$

(6) $$SD_{d}=\sqrt{\frac{\sum_{i=1}^{n}{\left(d-\bar{d}\right)}^{2}}{n-1}}$$

#### 2.3.2. Bland-Altman Analysis (Rigid Structure Tests Data)

A Bland-Altman analysis of agreement between EMA and a millimeter ruler were implemented to evaluate accuracy. To do that, all measurements obtained with the ruler were also determined from EMA data. For all 24 angular positions of the structure, the Euclidean distance between each pair of sensors was calculated from single samples using Equation (1). The points defined by the coordinates (x_1_, y_1_, z_1_) and (x_2_, y_2_, z_2_) correspond to the instantaneous position of two sensors, and d corresponds to the distance between them.

#### 2.3.3. Mandibular Movement Analysis (Model Tests Data)

An analysis of the SD of repeated measurements was implemented to assess the reliability of the articulograph when employed to measure distances associated with mandibular dimensions under static and dynamic conditions.

Instantaneous distances d_1_ and d_2_ Figure 2b were obtained from every recording of all tested conditions. The SD of those distances throughout every recording was calculated to be able to analyze the dispersion of the measurements under each condition tested.

## 3. Results

### 3.1. Trajectories Analysis

**Figure 3:**
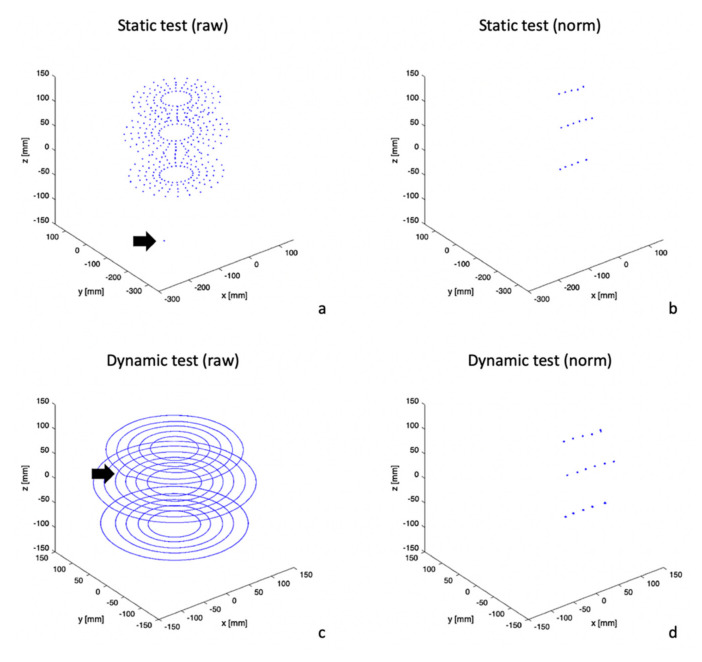
Trajectories of the recording set of each test. (**a**) Static test (raw). (**b**) Static test (norm). (**c**) Dynamic test (raw). (**d**) Dynamic test (norm).

The spatial representation of the raw data of the static test (Figure 3a) showed the static positions of all 16 sensors attached to the rigid structure (Figure 1a) in each of the 24 angular positions of the structure analyzed. The representation of the normalized data of the static test (from the same recording as the raw data but applying the “Head Correction” function) revealed 16 punctual markings—one for each sensor—with no angular variation (Figure 3b). The spatial representation of the raw data of the dynamic test (Figure 3c) showed circular trajectories performed by the 16 sensors. The representation of the normalized data of the dynamic test revealed similar characteristics as the normalized data of the static test, revealing only punctual markings with no angular variation.

The raw data spatial representation (Figure 3a,c) showed alterations on the upper section of the measurement area. Arrows in (Figure 2a,c) show that at an angulation of 195° the data shift unexpectedly. This effect was more intense for static tests than for dynamic tests, where a depression was observed in the circular trajectory (Figure 3c).

### 3.2. Reliability Analysis

#### 3.2.1. Position Determination

**Figure 4:**
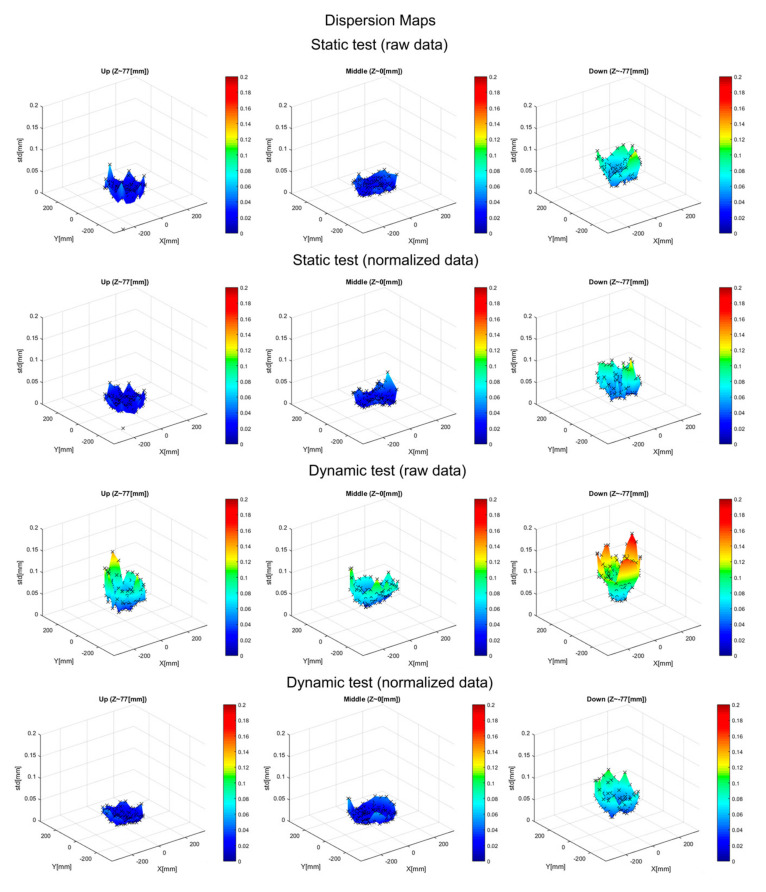
Position standard deviation (SD) maps for the repeated measurements registered in every test (rows from top to bottom: Raw data of the static test, normalized data of the dynamic test, raw data of the dynamic test, and normalized data of the dynamic test) for the three vertical levels considered (from left to right: Up, middle, and down). Black points indicate SD values calculated from registered data. The colored surface represents interpolated values included only to aid the interpretation of the graphs (the color bar indicates the SD level in millimeters).

Position SD maps (Figure 4) show the dispersion of the repeated measurements of the position of the sensors according to the location of the sensors. These maps reveal that the system reliability is higher at the centre of the measurement area and decreases progressively toward the edges. Position SD maps of the upper level for the static tests showed null values at an angular position of the structure of 195°, coincident with the unexpected shift of data detected on the trajectory plots. Position SD values fluctuated between 0.001 mm and 0.185 mm (including all tests and conditions but excluding the outlier at 195°).

#### 3.2.2. Distance Determination

**Figure 5:**
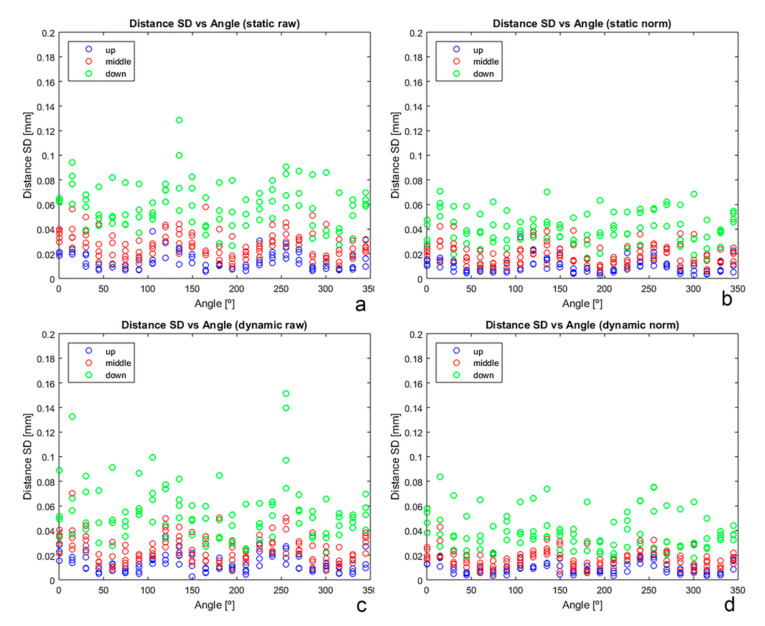
SD of repeated measurement of distances between pair of sensors versus angular position of the structure for all tests and conditions. (**a**) Static test raw data; (**b**) static test normalized data; (**c**) dynamic test raw data; (**d**) dynamic test normalized data.

SD of the calculated Euclidian distance between sensors showed that the AG501 precision diminishes downwards in the measurement area (Figure 5). There are also angular directions easy to identify in which the precision is better than in others. In all cases, dispersion was greater for raw data than for normalized data. Distance-measurement SD values fluctuated between 0.002 mm and 0.151 mm (including all tests and conditions but excluding the outlier at 195°).

### 3.3. Accuracy Analysis

**Figure 6:**
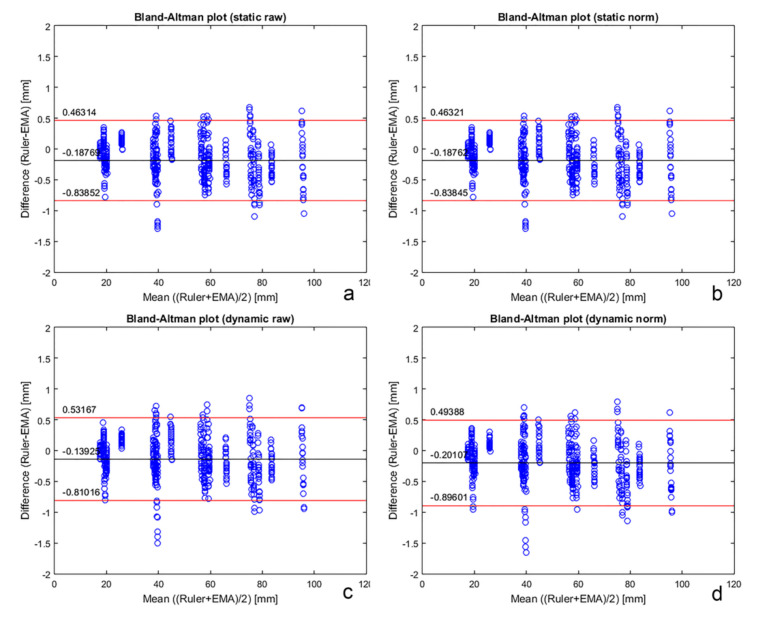
Bland-Altman plots to evaluate agreement between electromagnetic articulography (EMA) under different conditions and a millimetre ruler (black line: Mean value of the differences; red lines: Upper and lower limits of agreement). (**a**) Comparison between EMA raw data and the millimetre ruler measurements for the static test; (**b**) comparison between EMA normalized data and the millimetre ruler measurements for the static test; (**c**) comparison between EMA raw data and the millimetre ruler measurements for the dynamic test; (**d**) comparison between EMA normalized data and the millimetre ruler measurements for the dynamic test.

Bland-Altman analysis illustrates distance-measurement agreement between the AG501 and the millimetre ruler (Figure 6 and Table 2). Considering all cases, the maximal value for the upper limit of agreement was 0.5 mm, and the minimum value for the lower limit of agreement was −0.9 mm.

### 3.4. Performance on Mandibular Movement Registration

#### 3.4.1. Distance SD for Static and Dynamic Tests on the Mouth Anatomical Model

The SDs for the repeated measurement of distances between the sensors located on the mouth anatomical model for the static test and for the dynamic test are shown in Table 3 and Table 4.

#### 3.4.2. Distance SD for the Mouth Opening Test on the Mouth Anatomical Model

The SDs of the repeated distance measurements throughout all repetitions of the mouth opening test are presented in Table 5.

## 4. Discussion

Mandibular movements are studied in dentistry to diagnose problems associated with masticatory function, to program articulators for prosthetic restorations and to detect several temporomandibular joint alterations [23]. The evaluation of mandibular movements is highly relevant regarding functional evaluation of temporomandibular joints and a helpful indicator in diagnosis and treatment planning [24,25]. In studies that have mainly focused on interests of clinical dental evaluations, the lack of knowledge about the accuracy and reliability of Carstens EMA AG501 for mandibular movement analysis has been a main concern regarding the legitimization of this technology. Previous studies have evaluated EMA technology in articulatory phonetics, and most presented positive results [4,5,6,7,8,9,10]. However, there is still a lack of confirmation of how this technology behaves and could be applied to mandibular movement studies in dentistry.

For this purpose, a method to assess the accuracy and reliability of EMA applied to mandibular movement analysis was proposed and executed based mainly on the methodology applied in previous studies by Stella et al. and Savariaux et al. [9,10].

In addition to the methodology applied in previous studies [9,10], besides validation for mandibular movements of interest in dentistry, the present work introduces the analysis of data obtained after normalization and the implementation of the Bland-Altman analysis of agreement to assess accuracy [26].

A systematic error was observed in all 30 trajectory repetitions recorded during dynamic tests, and in the static test, this error occurred similarly at the 195° angle. In this case, normalization of the data acted to reduce this anomaly. Stella et al. also reported systematic anomalies in certain regions and with a specific pattern, as observed in the present study [8].

The reliability of the position recording of the sensors analysed by the deviation maps in the static and dynamic tests showed greater variations in the data in the dynamic tests. The analysis of the reliability of the sensors at different levels and positions showed that there was greater reliability in the central regions and that it decreased toward the edges of the measurement area. In addition, measurements were also more reliable at the higher level decreasing downwards. The precision modifications occurred systematically and congruently in the static and dynamic tests. The lower reliability of the EMA data during static tests found in the present study was also reported by Berry et al. in the NDI EMA system [6].

However, it is worth noting that even if differences in reliability were observed in different regions for both tests, in no case did the variation of positions exceed 0.2 mm, which is within the manufacturer’s acceptable values (dynamic positional accuracy of 0.3 mm RMS). These data corroborate the high reliability of EMA AG 501 seen in previous studies, which compared this equipment with other models of EMA systems [9,10].

The variation of the distance measurement between two points evaluated by the SD of the calculated Euclidean distance between the sensors showed that the precision decreases at the lower levels of the measurement region, maintaining the systematic behaviour previously observed in the dynamic and static tests for the position of sensors.

In all tests to assess precision discussed earlier, normalized data showed smaller variations compared with raw data. This might be due to a low-pass filter reported by the manufacturer. No other study evaluated this feature offered by the equipment, so it was not possible to compare the results obtained in the present study with the results of other studies.

The accuracy tests were performed comparing results with a millimetre ruler because it is the most commonly used instrument in dental clinical practice to evaluate ranges of mandibular movements, such as mouth opening and eccentric movements. The Bland-Altman statistical test applied to data from both measurement methods revealed an acceptable level of agreement, with maximum values not exceeding 0.5 mm and a minimum value of not less than −0.9 mm. Being within the resolution of the ruler (up to 1.0 mm). The EMA “Head Correction” function generated less impact on agreement levels when comparing precision. There were no major differences nor a definite trend in the comparison of raw versus normalized data.

Previous studies that evaluated the reliability and accuracy of EMA-based systems compared these systems against another method, which was considered the best interpretation of real values [5,6,7,8]. In the present study, the Bland-Altman analysis seemed more appropriate because it sought to estimate the agreement between the two methods evaluated, EMA and ruler, considering the inherent error of each method.

Tests to assess precision of the distance measurement between two points on the mouth anatomical model showed that there were differences when the model was placed in different positions, which simulated extreme clinical possibilities.

In all tests with the mouth anatomical model, the values of the variation of the distance measurements between two points were within those reported by the manufacturer. For distance measurements, the sum of the error inherent to the positional tracking of each sensor (0.3 mm RMS) was considered, resulting in a final error of 0.6 mm for distances. The normalization of the data subtly modified the values obtained from static tests.

To date, there are no previous studies performing a similar analysis of mandibular movements. The data presented in the present study suggest that even if patients are in extreme positions of flexion, extension, or rotation of the head and neck, the EMA will be able to evaluate positions and distances with variable errors, but in all cases within the values given by the manufacturer.

One limitation of this study is the disadvantage of using the mouth anatomical model, as it does not contain the soft tissues and their associated humidity and also does not perform movements with the same complexity as the oral cavity of a person. However, the present work proposes the use of a mouth anatomical model because of its better measurement standardization.

In the present study, the data obtained from the tests with the EMA AG501 had high accuracy and reliability. The higher and more central regions of the measurement area showed greater accuracy, and the static evaluations were more accurate than the dynamic ones. However, in all tests, the variations of these values were within the range of variation given by the manufacturer. The normalization of the data through the “Head Correction” function softened these variations of precision in the dynamic and static tests; however, it did not have a great impact on the reliability. Static and dynamic tests using the mouth anatomical model revealed the same high reliability, and these data showed that the equipment resulted in accuracy within that reported by the manufacturer, even when extreme positions of the head and neck were simulated. These characteristics suggest that the EMA AG501 is valid for 3D analysis of mandibular movements because of its high accuracy and reliability, and because it does not contain structures in arches or a large structure attached on the head, it allows for more natural movements by not immobilizing any part of the head or neck. This makes this technology highly recommended for research purposes. Its application would improve three-dimensional analysis of mandibular movements. Further investigation is needed to determine whether or not it should be considered for clinical use or real life domain of dentistry. One considerable caveat about this method is the possibility of occurrence of systematic errors, as observed in the present study and reported in previous studies. Therefore, it is suggested that each research group test the occurrence of these errors in their equipment and avoid measurements in conflictive areas.

## 5. Conclusions

With the limitations of this in vitro study, the results suggest that EMA AG501 is valid for three-dimensional analysis of mandibular biomechanics allowing natural movements.

## Figures and Tables

**Table 1:** Tests conducted.

Structure Used	Type of Test	Active Sensors	N	Data Analyzed
Rotating structure	Static	16	30	Raw and normalized
	Dynamic	16	30	Raw and normalized
Mouth anatomical model	Static	3	30	Raw and normalized
	Dynamic	3	30	Raw and normalized
	Mouth opening	3	30	Raw and normalized

**Table 2:** Bland-Altman EMA vs. ruler.

	Static Test		Dynamic Test	
	Raw Data	Normalized Data	Raw Data	Normalized Data
Mean of diff. (mm)	−0.19	−0.19	−0.14	−0.20
SD of diff. (mm)	0.33	0.33	0.34	0.35
Limit of agreement max (mm)	0.46	0.46	0.53	0.49
Limit of agreement min (mm)	−0.84	–0.84	−0.81	−0.90

**Table 3:** Distance SD (mm, static test).

Position	Raw Data	Normalized Data
0°	0.04	0.02
90° Extension	0.05	0.03
70° Flexion	0.37	0.37
78° Left rotation	0.27	0.27
78° Right rotation	0.03	0.02
78° Left flexion	0.41	0.41
78° Right flexion	0.24	0.23

**Table 4:** Distance SD (mm, dynamic test).

Movement	Raw Data	Normalized Data
90° Extension	0.58	0.58
70° Flexion	0.48	0.48
78° Left rotation	0.18	0.18
78° Right rotation	0.39	0.39
78° Left flexion	0.37	0.37
78° Right flexion	0.38	0.38

**Table 5:** Distance SD (mm, mouth opening test).

Movement	Raw Data	Normalized Data
0°	0.06	0.05
90° Extension	0.13	0.13
70° Flexion	0.13	0.13
78° Left rotation	0.14	0.14
78° Right rotation	0.23	0.23
78° Left flexion	0.25	0.25
78° Right flexion	0.12	0.12
